# Limb Prostheses: Industry 1.0 to 4.0: Perspectives on Technological Advances in Prosthetic Care

**DOI:** 10.3389/fresc.2022.854404

**Published:** 2022-03-10

**Authors:** Silvia Ursula Raschke

**Affiliations:** British Columbia Institute of Technology, Applied Research, MAKE+, Burnaby, BC, Canada

**Keywords:** prosthetics, technology, Industry 4.0, innovation, history

## Abstract

Technological advances from Industry 1.0 to 4.0, have exercised an increasing influence on prosthetic technology and practices. This paper explores the historical development of the sector within the greater context of industrial revolution. Over the course of the first and up the midpoint of the second industrial revolutions, Industry 1.0 and 2.0, the production and provision of prosthetic devices was an *ad hoc* process performed by a range of craftspeople. Historical events and technological innovation in the mid-part of Industry 2.0 created an inflection point resulting in the emergence of prosthetists who concentrated solely on hand crafting and fitting artificial limbs as a professional specialty. The third industrial revolution, Industry 3.0, began transforming prosthetic devices themselves. Static or body powered devices began to incorporate digital technology and myoelectric control options and hand carved wood sockets transitioned to laminated designs. Industry 4.0 continued digital advancements and augmenting them with data bases which to which machine learning (M/L) could be applied. This made it possible to use modeling software to better design various elements of prosthetic componentry in conjunction with new materials, additive manufacturing processes and mass customization capabilities. Digitization also began supporting clinical practices, allowing the development of clinical evaluation tools which were becoming a necessity as those paying for devices began requiring objective evidence that the prosthetic technology being paid for was clinically and functionally appropriate and cost effective. Two additional disruptive dynamics emerged. The first was the use of social media tools, allowing amputees to connect directly with engineers and tech developers and become participants in the prosthetic design process. The second was innovation in medical treatments, from diabetes treatments having the potential to reduce the number of lower limb amputations to Osseointegration techniques, which allow for the direct attachment of a prosthesis to a bone anchored implant. Both have the potential to impact prosthetic clinical and business models. Questions remains as to how current prosthetic clinical practitioners will respond and adapt as Industry 4.0 as it continues to shape the sector.

## Introduction

The term Industry 4.0 is frequently used enthusiastically to describe a new wave of exponential innovation that will revolutionize the world as we know it and, with it, the field of orthopedics, including prosthetic devices (1, 2). The term itself signals it is not the first, inviting the questions “What are the industrial revolutions?” and “What do they have to do with prosthetics?”

It has become common to describe technological evolution within a framework of industrial revolutions, which are used to denote eras of significant change in how goods are designed and produced or how technological developments change products and processes. Initially it was used to describe the transition from an agrarian society to an industrial one beginning in the mid 1700's. It is now used to describe four eras: The Industrial Revolution (Industry 1.0), The Technological Revolution (Industry 2.0), The Digital Revolution (Industry 3.0) and the Physical, Digital, and Biological Convergence (Industry 4.0) (2, 3).

This paper aims to present examples from the history of prosthetic technology evolution within the industrial revolution framework to highlight how current prosthetic provision clinical practice and business models could benefit from current technological innovations or could be significantly disrupted by ^1^ it. It is hoped this paper will help current prosthetic service providers understand the need to be proactive in navigating these cross-sectoral changes as the engineers and technology developers driving them begin to insert themselves into the prosthetic provision process.

It should also be noted that the history presented focuses predominantly on developed nations, as the use of technology intensive prosthetic componentry is still concentrated in developed nations. However, Industry 4.0 is seen as having the potential to improve access to care globally. Initial improvements to access to care are already emerging in response to the Covid-pandemic (4). Specific to prosthetics, additive manufacturing to improve access to prosthetic technology in low- and middle-income countries is seen as an emerging area of research and development (5).

**Figure 1:**
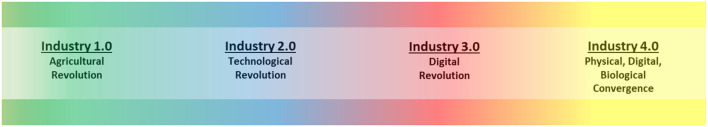
Industrial innovation as a spectrum with no fixed points between eras.

This paper traces the four industrial revolutions, identifying key themes and presenting some of the innovations that occurred in each era that eventually found their way into prosthetic design and practice. There are limitations to this approach, as each era does not have clearly defined start and end dates. And, because of the lag in adoption of new technologies and processes by the sector, along with non-liner technology development, it is perhaps better to think of progress in this sector as a spectrum with some overlap between eras (Figure 1).

It is in no way a complete history of either industrial revolutions or the prosthetics sector. Instead it seeks to link how the key themes of each era of industrial revolution eventually impacted, in some way, prosthetic design or practice. It also explores how technological change and historic events shaped the prosthetics sector actively, embracing innovation or change, as well as passively, where innovation or change occurred because it was no longer possible to maintain the past way of doing things.

## Industry 1.0: Industrial Revolution

Industry 1.0, also known as the First Industrial Revolution or simply as the Industrial Revolution, began with the development of mechanisms to harness water and steam power to drive industrial machines in the eighteenth century. This period of industrialization shifted society's focus from agrarian to industrial giving rise to machinery that could produce goods that up to that point had been produced by hand. This was done in factories that, coupled with improved and more efficient transportation, allowed goods to be moved further and more cost effectively than before (3). Classic examples are water powered looms used to weave cloth in mills and steam trains that transported people and goods to factories and markets. Agriculture was also becoming mechanized, allowing more efficient food production and freeing farm workers to move to larger centers to work in mills and factories. This was not a revolution in the military sense. It was a revolution in the way people lived, worked and conducted business. At the start of Industry 1.0, the production of prosthetic limbs was craft based using locally available materials such as leather, wood and metal (6–9). Amputation was typically due to trauma which was often the result of warfare and few amputees could afford a device. Prosthetic limbs were not widespread and amputees improvised with what they had in order to ambulate. Prior to and including the period spanning Industry 1.0, little to no advancement in prosthetic design occurred over a period of centuries.

## Industry 2.0: Technological Revolution

Industry 1.0 began to transition to 2.0 at the mid to latter part of the nineteenth Century with some sources marking the First World War as the start of the Industry 2.0 (10). This period was marked by the invention of devices that could capture and store electrical energy leading to electrification of factories and an increasing use of mass production. At the same time many novel materials and inventions were created. Industry 2.0 is commonly referred to as the Technological Revolution.

Over the course of Industry 2.0 two wars left their mark on the prosthetics sector, leading to the establishment of the practice of prosthetics as a specialty and providing the first significant innovative impulse in prosthetic device design and production. In parallel, a number of technological and societal changes took place that did not immediately influence prosthetic design and practice, but which laid the groundwork for later significant changes in the sector.

### The Great (US) Civil War Benefaction and the Emergence of the Prosthetic Specialist

The first of the wars, the American Civil War, left a large number of amputees in its wake and a recognition that these Veterans should be provided with some form of prosthetic device that they did not have to procure at their own expense. This led to a financial commitment by the US Government to provide all veterans with prosthetic devices through what was know as the Great Civil War Benefaction (11). This in turn generated a burst of prosthetic technology development activity, resulting in numerous patented prosthetic designs and represents the first time in history that “Industrial” thinking had been applied to creating solutions for what had previously been an *ad hoc* approach to prosthetic design and production. One of those inventors, an engineer, Civil War veteran and amputee, J.E. Hanger, not only patented innovative prosthetic designs, but also established the J.E. Hanger prosthetic workshops where employees specialized solely on making and providing prosthetic devices, as opposed to such devices being made by metalworking trades alongside other items such as horse shoes or tools and other implements. This was a pivotal shift in production and delivery model and with the ability to concentrate exclusively on^2^ ^3^ ^4^ the production of one type of device, standardized approaches and further design refinements began to emerge, leading to the establishment of prosthetics as a recognized, stand-alone trade.

### Physical Therapy for Amputees and Modular Prosthetic Systems

The second of the wars, the First World War, created more than 41,000 amputees in Britain alone. In treating this large cohort, the first connections began to be made between amputation and the psychology of limb loss, changing how amputation and amputees were viewed in society (12). Physical therapy and gait training became part of the post-amputation recovery process for the first time. This wholistic approach to amputation led to an evolution of the construction and appearance of prosthetics limbs, including the reimagining of the prosthesis as a functional tool where terminal attachments resembling industrial equipment took the place of the prosthetic hand, as opposed to the prosthetic hand being an imperfect cosmetic replacement that provided some basic functions. The concept of the prosthesis as an industrial, functional tool disappeared by the 1950's, but foreshadowed the more radical reimagining of the prosthetic limb that was to come under Industry 4.0 (13).

In Germany the need for prosthetic devices for war amputees was also immense, sparking the development of a modular prosthetic system using standardized, mass produced components very much in the spirit of industrial revolution. Developed by the prosthetist Otto Bock, this innovation created a novel paradigm for prosthetic device production that assembled prostheses from components that had been designed, manufactured and quality tested and that were then attached to the socket, which was still hand-crafted. This allowed work processes to be rationalized, improving the efficiency of the prosthetist and providing a guarantee of safety and quality that is not possible to provide for hand crafted components (9). From this point onward, though still be done in practice, it was no longer *necessary* to hand craft prosthetic components other than the socket interface with the residual limb.

### Early Human Movement Studies, Materials, and Electronic Innovations

While not applied to prosthetics at the time, developments in other sectors created innovative building blocks that would be applied to prosthetics technology and practices later, as Industry 2.0 gave way to Industry 3.0 in the 1960's.

One such building block was technology and processes which supported the study of human and animal movement. Interest in movement of the body dates back as far as the Renaissance, but it was the invention of the camera that allowed the first quantitative biomechanical studies to be done by Etienne-Jules Marey, using photos. Carlet and Muybridge followed, using early pressure recording shoes and film respectively (14). Over the course of Industry 2.0 these tools were refined and physiological monitoring technology such as VO_2_Max was introduced. These were integrated into movement and gait analysis systems, which were then applied to the study of amputee gait and prosthetic device design beginning in the 1960's (15).

A second set of building blocks emerged in the chemical sciences from the 1870's to the 1930's, beginning with the study of natural resins and polymers and leading to the development of synthetically manufactured resins. From this a range of new materials, related processes and resulting products emerged (16). In the biomedical sciences, dentists were early adaptors of these materials during this period, using them for fillings and restorations. These materials did not begin to find their way into prosthetic limb production until the late stages of Industry 2.0 in the 1960's, when they were used to create both the outer cosmesis of prosthetic limbs and replacing hand carved wood sockets with composite laminated sockets (17).

A final example of building block innovation in this era is early work on myoelectric control, which began during the Second World War. The first known reference is a patent application for myoelectric control of a prosthetic hand made by Reinhold Reiter in Munich, Germany (18). This was followed by a rapidly growing body of research harnessing transistors to create upper limb myoelectric prostheses, work that was being pursued independently and collaboratively across the globe, including efforts in Japan, the US, Italy, Germany, Canada, UK, Russia, Sweden and Austria. These early digitally-controlled arms laid the groundwork for the use of micro-processors in upper limb, and later lower limb, prosthetic devices under Industry 3.0 (19).

## Industry 3.0: The Digital Revolution

Industry 3.0 is characterized by inventions such as the transistors, processors and computers which became smaller, more powerful and more flexible as the technology was refined and improved. Beginning sometime between 1950 and 1970 (3), and running until ~2010, it ushered in a wide range of increasingly sophisticated prosthetic designs and components that were developed using interdisciplinary approaches. Human movement research was used, for the first time, to evaluate prosthetic design and function in a wide range of studies. This was a significant departure from previous prosthetic design practices where advances based on personal experiences were considered to be “trade secrets” to be passed down the generations and were not independently and objectively evaluated for function or effectiveness.

### Digitization

Computers and digital technology became ubiquitous in workplaces and home during this era as desktop computers gave way to laptops, tablets and phones. This was supported by the development of cellular telecommunications, the Internet, Wi-Fi and Bluetooth, creating the ability for computing technology to follow the user wherever they went. Software was developed to run on the wide range of resulting hardware platforms, profoundly impacting the design and manufacturing sectors through tools such as Computer Aided Design and Manufacture (CAD-CAM), Flexible Manufacturing Systems (FMS) and Advanced Digital Manufacturing (ADM). This set the stage for disruption of prosthetic production under Industry 4.0.

Beginning in the 1980's digital design and manufacturing tools developed for other sectors began to make their way into the prosthetics sector. The application of CAD/CAM to the production of prosthetic devices was a natural offshoot of the success of CAD/CAM in other fields, adding efficiencies and reproducible accuracy. Early champions identified multiple benefits coming from the adoption of this technology in prosthetics (20). Seminal work was done to create sector specific software and hardware and included CANFIT (Vorum), Seattle ShapeMaker and CAPOD systems, with Vorum becoming an industry mainstay internationally (21).

Digitally supported advances were not limited to production processes. The far more visible and impactful digital transformation occurred in prosthetic componentry, setting new standards for prosthetic device function and end-user outcomes. Digital solutions first made their mark in upper limb prosthetics early in this period but it was in lower limb prosthetics that digital technology had the most perceptible impact on amputees, with the introduction of microprocessor controlled (MPC) joints. The Intelligent Knee (Blatchford Ltd., 1990) became the first MPC prosthetic knee to enter the marketplace quickly followed by the C-leg (Ottobock Gmbh, 1997) and then, MCP ankles and feet. These components profoundly changed the amputee experience by addressing functional needs and safety that previous prosthetic designs and technology were unable to (22–25).

### Materials Sciences and Collaboration

Digital applications are the hallmark of Industry 3.0, but it was the adoption of synthetic polymers developed during Industry 2.0 most visually changed prosthetic technology in the early days of Industry 3.0. At that time prosthetic devices were still carved from wood, forged from metal and completed with customized leatherwork. New materials quickly transitioned the sector away from those materials to acrylic and polyester laminates. Though not “digital” in nature, the adoption of these new materials supported a paradigm shift that allowed novel prosthetic designs to be developed using structured processes incorporating interdisciplinary criteria, such as biomechanics and anatomy, into the design process (26). The move from carefully guarded “trade secrets” as the basis of prosthetic design to the use of objectively validated design iterations had begun.

Following World War 2, university research programs supporting improvements in prosthetic design were initiated. Universities began to influence the sector with developments such as the Supracondylar Socket, Patellar Tendon Bearing Socket, Four-Bar prosthetic knee joint mechanism, SACH (Solid Ankle Cushion Heel) Foot and Seattle Foot, each of which capitalized on materials sciences advances that were now coupled with structured engineering design practices and were carried out within an academic environment.

Collaborations between private industry and academic or public institutions also contributed, the classic example being the myoelectric arm combining the use of new materials with digital technology and developed by Ottobock Gmbh in collaboration with institutionally based research programs, such at the ones at I.N.A.I.L in Italy (18). A second example is the development of silicon liners as an alternative to cotton and wool stump socks and which provided additional benefits to amputees such as improved comfort and performance as well as providing suspension. Silicon liners, first developed in industry by Össur, were validated in scientific studies carried out at universities (27, 28).

### Biomechanics Emerges as a Specialty and the Emergence of Clinical Outcome Measures

The objective study of prosthetic gait today cannot be imagined without digital technology. The integration of cameras capturing 3D movement with force sensors launched kinematic (motion) studies and kinetic (force) studies as a formal area of research. Leaders in applying this to pathological gait and prosthetic applications are Inman (UCLA-SF) and Perry (Rancho Los Amigos National Rehabilitation Center), the latter of whom expanded the tool kit by adding fine wire electromyography. Professor Paul aided, by Jarrett and Andrews, was instrumental in digitally integrating these tools, setting the stage for modern gait analysis systems (14, 29). These pioneering researchers built the foundation upon which laboratory-based research quantifying prosthetic gait is carried out to this day, allowing the examination of how prosthetic design influences amputee function and considering of how the resulting knowledge can be translated into clinical practice.

As the digital revolution gained momentum, the range of digital tools expanded to include scanners to capture residual limb shapes for modification in CAD/CAM systems, step count monitors allowing the tracking of community-based activity of prosthesis users (30), and tools to aid in objective alignment of prosthetic devices such as the 3D L.A.S.A.R. Posture (Ottobock), Compas and Smart Pyramid (Orthocare Innovations) (31, 32).

The development of these tools provided new perspectives on the prosthetic device provision process. Their objective, valid and reliable evidence-based outcome measures presented an alternative to the subjective “clinical expert” opinion that, up to that point, had been the standard for determining if prosthesis fit or function was satisfactory, or not. Despite their availability, uptake of the new digital tools was slow on the part of prosthetic practitioners in large part because they did not provide direct benefit to the clinical practitioner by improving efficiency, increasing productivity or boosting the bottom line. Compounding this, many of the new tools had high entry costs which proved to be a tangible barrier to adoption (33).

In the academic setting, the development of clinical outcome measures specific to prosthetics gained momentum in the mid 1990's. In particular, Gailey (University of Miami) and Hafner (University of Washington) were carried out critical, objective research on amputee gait that led to the development of a wide range objective clinical evaluation measures including, but not limited to, the AMPRO, AMPnoPRO (Gailey) and Plus-M (Hafner) (34, 35).

The need for such measures had already been identified in literature by Ramstrand and Brotkorb (36) but until the publication of the Levinson Report in 2011 (37), discussed in the next section, the audience for this growing body of research was limited to the academic setting. Prosthetic device providers still took much pride in their “hands on” experience-based knowledge often speaking of seeing “with their hands” during this latter phase of Industry 3.0 (9). Prosthetists have been slow to voluntarily adopt even simple clinical outcome measures for a range of reasons including a lack of the time it takes to carry the out an a lack of clarity as to the value they measures provide (38).

The entry costs for digital tools in this sector have significantly reduced over time but, in developing technology for this sector, this stage of prosthetic history illustrates the importance of balancing the full spectrum of economic costs vs. benefits; a critical factor in prosthetics due to the highly cost sensitive nature of the fee-for-device business model.

### Adoption of Evidence-Based Practices and Clinical Outcome Measures in Clinical Prosthetic Practices

Clinical outcome measures and evidence-based practices were not seriously considered in the clinical setting until the publication of the aforementioned Levinson Report (37) by the US Department of Health & Human Services' Office of the Inspector General. The report was highly critical of prosthetic billing practices within the Medicare system in the United States, exposing a structural vulnerability in the sector, namely the lack of ability to demonstrate cost-benefit using objective criteria. It was a watershed moment, allowing insurers to require justification for reimbursement to be supported by objective, measurable outcomes as opposed to subjective expert clinical opinion or experience.

This report set in motion the translation of academic research on clinical outcome measures and digital evaluation tools practices into the clinical setting. Momentum was built by the active support for the development of evidence-based practices and tools through funding from organizations as diverse as: American Academy of Orthotists and Prosthetists (AAOP). American Orthotics and Prosthetics Association, the U.S. National Institutes of Health (NIH), U.S. Department of Defense (DoD) and the Orthotics & Prosthetics Education and Research Foundation (OPERF). This established an ongoing collaborative, interdisciplinary effort that is creating evidence-based knowledge on clinical and technical issues that relate to amputees and prosthetic care, including the use of digital tools.

## Industry 4.0: Physical, Digital, and Biological Convergence

The term Industry 4.0 describes a convergence of Physical, Digital and Biological Systems that support the creation of “smart” technology or cyber-physical systems. The resulting technology can be networked and allows for the collection and storing of large amounts of data in data bases which have value in themselves as drivers of innovation.

Smart technology is often described as disruptive, spanning nine enabling technologies: Advanced Manufacturing, Additive Manufacturing, Data Analytics, Augmented Reality, Simulation, Horizontal/Vertical Integration, Cyber Security, Cloud Computing and the Industrial Internet.

**Figure 2:**
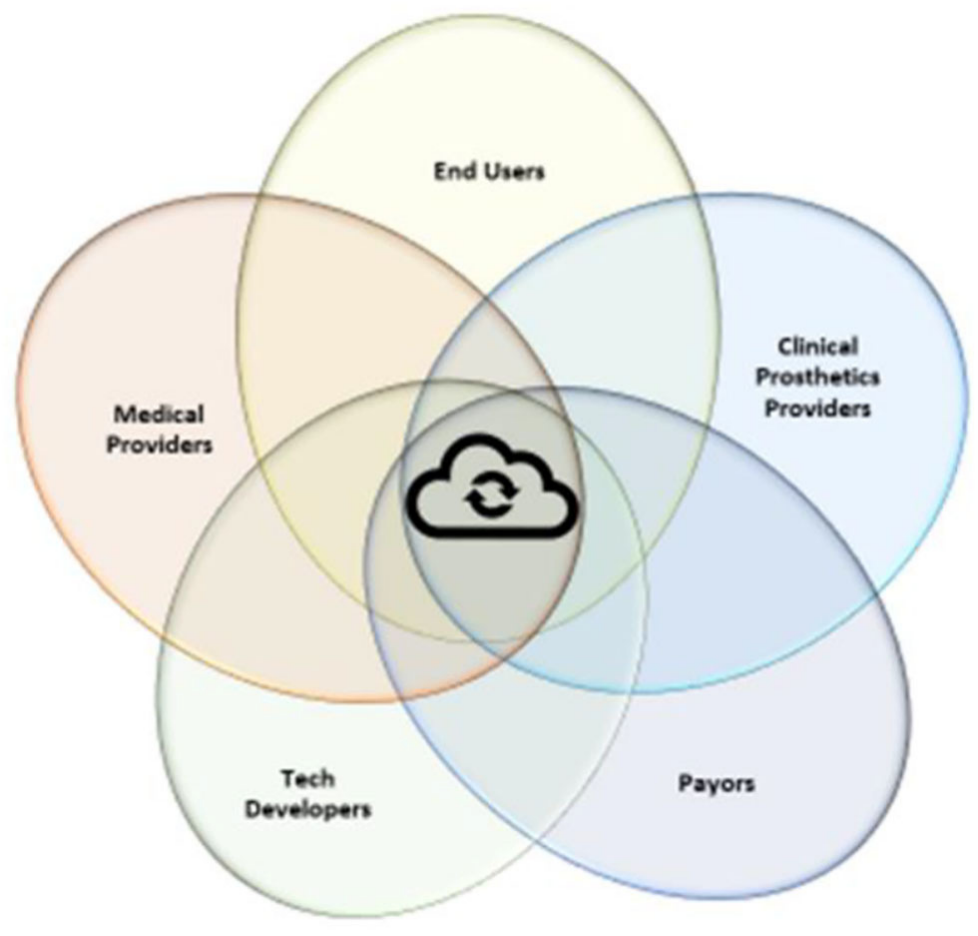
Innovation will take place as various experts and bodies of knowledge co-create synergistically through the sharing of expertise and knowledge where they overlap with the other fields. The hub of this sharing will be data sharing.

At first glance this list has little relevance to the day to day production and provision of prosthetic devices but, as was the case in Industry 2.0 and 3.0, the prosthetics sector will be influenced and shaped by Industry 4.0. Technologies from this list that have already begun to manifest themselves in the prosthetics sector include Additive Manufacturing (3D printing), Smart Sensors, the Internet of Things (IoT), Blockchain, Software as a Service (SaaS), Machine Learning (M/L) and Big Data (39). In the prosthetics sector innovation will no longer be propelled by individual discoveries or events, such as a new material, but by overlapping influences that intersect and act synergistically at a data driven hub (Figure 2).

### Socket Evolution: Materials Sciences and Machine Learning (M/L)

As in the previous industrial revolutions, advances in materials sciences are playing a prominent role in the evolution of prosthetic devices under Industry 4.0. In previous eras materials advances led to improved cosmesis, function and fit but the general form and design of prosthetic devices remained the same. Under Industry 4.0 data driven engineering approaches are being applied to materials development. This is allowing prosthetic designs metamorphosize and, in particular, there is now the ability to address shortcomings that have been identified with the classic prosthetic socket (40).

Changes in volume of the residual limb have been a long-identified problem with classic, rigid socket designs. Past approaches to managing residual limb volume fluctuation ranged from early adjustable sockets made of leather and lacers (41, 42) to adding and taking off stump socks over the course of the day, the latter of which is still done today but which is not a wholly satisfactory solution (43). Research has begun to point to the current standard of care being inadequate and thoughtful approaches are beginning to emerge (44). As in past eras, these draw on an expanding palette of materials alongside seeking more widely sourced innovative elements, for example the sports equipment and high-performance garment sectors and then combining them in more complex and novel ways. These adjustable socket solutions allow amputees to easily adjust socket volume manually over the course of a day eliminating the need to don and doff a prosthesis or to readjust laces, both of which take time and effort and require direct access to the prosthesis (45). Manually controlled designs have given amputees improved control over the fit of their sockets, but this is only the beginning. Early work on automated adjustable socket designs has started, creating a potential future where socket fit is adjusted automatically in close to real time (46). The added benefits of these engineering and technology-based developments are that they will allows for greater quality control in the production of the socket itself, reducing product liability risk and potentially creating efficiencies within the production and provision processes. This example is one which illustrates the increasing complexity of solutions and how ideas and components from across sectors can now be combined to become more powerful than in previous eras.

At the complex end of the spectrum tools such as 3D Printing will be supported by new processes for measuring residual limbs using smart phones, scanners and other imaging technology. Highly sophisticated and complex methods of objectively capturing surface anatomy, images of the underlying anatomical structures and potentially, pressure gradients (47) and tissue properties, will be combined with data bases of anthropometric and biomechanical measurements to which machine learning (M/L) will be applied and used to generate custom designed sockets (48, 49). 3D printing will allow integration of added value elements into final product, for example through the use of copper infused filaments with antimicrobial properties (50). The personalization of devices will be further informed by 3D motion data collected not only by researchers in prosthetics but also those from the physical and exercise therapy fields, such as that being used in the development of automated active assist devices to support rehabilitation (51). This is an interdisciplinary, wholistic re-imagining the prosthetic design and provision process and removes the last subjective step, the creation of a traditional socket, from the prosthetic production chain, making it theoretically possible for the entire prosthesis to be generated from objective design criteria using quality-controlled production methods. Fully automated socket design and production process may not ultimately be desirable as end-users will likely always wish to have and will benefit from having an expert assess prosthesis fit and function, but by using more data informed approaches in the creation of the socket the prosthetic provider will have a more objective baseline to begin an optimization process from.

### Smart Garments and Smart Technology

Smart garments combine novel fibers and textiles with sensors and data streaming capabilities. They can be used to monitor and diagnose medical conditions or, by the very nature of their properties, provide benefits to the wearer including equal or superior clinical outcomes, lower costs or better customer experience. Digital tools integrated into garments will become commonplace throughout the entire health care system and, in addition to enhancing care, will allow vast data bases to be created and mined to support decision making processes.

Smart materials have already entered the orthotics sector. Garments such as the Stoko Leggings and the DM Orthotics Scoliosis Garment harness properties created by the way a yarn is composed and spun or how the garment's components are combined in order to provide benefits that are novel or replace a more complex, and sometimes costlier, orthotic device (52). Smart garments will find their way into prosthetic designs as well, in the form of socket liner systems, control systems and clinical assessment tools. One garment, Hexoskin^tm^, has been validated as a tool for collecting physiological measures for a range of activities (53), including walking and could conceivably be built into protocols for evaluating prosthetic function (54). Smart technology will allow sockets to become an active component of the overall prosthesis contributing to improved fit and function (55), much like the MPC knees and feet did when they first became available. “Smart” sockets will integrate sensors that monitor pressure, fit and temperature (56), and will eventually be able to respond independently and dynamically to an amputee's physiological state or activity, all whilst streaming collected data into data bases.

Socket liners will become active monitoring and data collection components that complement microprocessor-controlled components at the knee, ankle, elbow or wrist. Data bases resulting from smart sockets and liners will be cross referenced with existing anthropometric and biomechanics data bases to which machine learning (M/L) and generative design practices will be applied (57). This will support the development of components that allow more complex and natural movement (58) and which will integrate sensors that enable temperature, touch and pressure to be incorporated into local feedback loops. Other research, focusing on implantable neural interfaces and brain-controlled interface (BCI), aims to allow the integration of BCI into prosthetic designs to drive prosthetic component control systems and provide real-time neural feedback concurrently (59).

The prosthesis of the future will be one that is custom designed and produced for individual end-users using objective design tools and automated industrial production methods and will be fitted and maintained using smart tools that provide objective, close to real time, data. This will allow the prosthetist to complete the evolution from being a crafter and fitter of devices to become a clinical technology manager, in partnership with amputees.

### Automation, Apps and Software-as-a-Services (SaaS)

In this transformation to becoming technology mangers, automation and Software-as-a-Service (SaaS) can assist prosthetists with improving quality and outcomes, whilst at the same time addressing productivity and labor shortages, another challenge currently faced by the sector and which will require the adoption of new approaches and technology.

The World Health Organization (WHO) identified a lack of skilled personnel at the international level. The National Committee on Orthotic and Prosthetic Education (NCOPE) has identified a labor shortage in the US (60), a prosthetic services review is underway in the National Health Service (NHS) in England (61), and the topic emerged anecdotally in Germany during data collection by Seibt in his study of how Industry 4.0 was changing the prosthetics sector (9). This is clearly a global sectoral challenge.

In sectors with labor shortages, including health care service delivery, automation processes and the use of AI can help ameliorate workload and productivity challenges and at the same time improve clinical outcomes (62). Automation as part of the solution has become a controversial one and is often met with fear and resistance, in particular where the transition to an “automated” data-based design and production process is being introduced to sectors that still engage in hands-on, craft-based production.

One of two perspectives on automation typically surface when discussing Industry 4.0. One provocatively presents the automated “robot” as a replacement for the worker. The other presents automation as a tool to help improve productivity and quality (63). The former narrative preys on the fear of change but is unlikely as robots and other forms of automation have limitations and are best used to replace repetitive and predictable tasks. They will become more flexible and applicable to a wider range of uses as their development matures and integrates artificial intelligence (AI), but it is highly unlikely, even in the long-term, that robots will replace the prosthetic clinician.

Efforts to move the prosthetics business model from fee-for-device to fee-for-clinical services have met with limited success globally. The prosthetics practitioner is increasingly challenged to find efficiencies within their current business model. This is where automation tools will be able to play a positive role, by allowing prosthetic practitioners to restructure their prosthetic design and production activities improve productivity. This is no a scenario in which robots take over. This shift will happen in parallel with software advances that improve the efficiency of clinic practices by re-shaping current administrative and business practices. Much of the focus in the prosthetics sector has been on hardware related innovations, but software innovation will have an equal impact. Two are worth exploring within the context of this paper, Apps and Software-as-a-Service (SaaS).

App is short for Application, a small piece of software designed to carry out a specific task using smart phones, tablets and other digital devices. Apps are already available in the prosthetics sector supporting tasks such as taking outcome measures, aligning, tuning or monitoring a prosthetic device, taking scans of body parts, interfacing with electronic medical records (EMRs) and providing a portal for communicating with payors (64). Apps are also empowering end-users by allowing amputees to self-manage their conditions across a range of situations from controlling MPC componentry to monitoring glucose levels. They can also support interactive and wholistic care models by enhancing communication and relationship building with clients, an example being the Ottobock Gmbh. fitness app. Finally, apps can provide a portal to Software-as-a-Service (SaaS) to support communication and interactions with central fabrication facilities, assist with the product design and provision process and in facilitating business transactions.

SaaS consists of subscription-based software platforms that support a range of business, production and clinical activities. SaaS reduces the initial cost of purchasing software and the associated costs of maintaining software in house. It can assist a clinical practice to become leaner, aiding with billing and reporting to payors, communicating with clients, organizing clinical outcome measures and tracking quality assurance data. It can also support liability management processes and can help maintain business continuity in crisis situations. SaaS will become an essential component of the prosthetic business model of the future, working synergistically with the manufacturing software and hardware. Finally, SaaS's networked nature will allow for prosthetic clinics to network with others that use the same platform(s) in order to collaborate to create large data bases that can be shared and mined, helping network members to maintain a collective competitive edge.

If harnessed strategically, these tools can allow prosthetists to reduce the time they devote to either producing prosthetic devices or wrestling with administrative tasks, allowing them to reallocate that time to focusing on their relationships with their clients, the amputees.

### Medical Treatment Advances

Industry 4.0 will also be accelerating advances in medical treatment options *via* the new tools being created and by using modeling, machine learning and AI to harness data bases. It is difficult to predicted what the impact will be as these developments are still in the early to mid stages of the innovation pipeline. But two examples with the potential to disrupt current prosthetic practices have matured and are translating to the clinical setting in developed nations.

The first is in the treatment of diabetes, which creates a high societal burden and is a common cause of amputation in developed nations (65, 66). A wide range of approaches to improving the management and treatment of diabetes is being pursued, crossing the spectrum from apps to allow diabetics to better manage their condition, for example by tracking what they are eating to medical device-based approaches such as continuous glucose monitoring and insulin pumps, which sit alongside surgical innovations such as islet transplantation. Finally, there is the emergence of personalized medicine approaches (67), with a large-scale Swedish study recently reporting improved health in Type 1 diabetics, including a 40% decline in amputation, when using a personalized medicine approach (68). These multimodal and technical advances are encouraging for diabetics and while reduced amputation rates indicate success, this will reduce the number of lower limb prostheses required in developed nations, which in turn will impact current prosthetic business models.

The second, specific to prosthetics, is osseointegration (OI) or bone anchored prostheses which sit directly on the intersection of the Physical, Digital and Biological as a medical-technological hybrid solution for existing amputees. OI involves attaching prosthetic leg componentry direct to a bone anchored implant, much like dental implants work. This eliminates the prosthetic socket completely and with it, many of the problems associated with the fit and use of sockets. OI provides the additional benefit of providing a secure interface between prosthesis and skeleton, which has been shown to improve osseo-perception and walking ability (69). It does not come without its own risks, such as implant loosening or failure or infection and skin irritation at the stoma, but international experience has shown it to be a viable option, even preferable, for some amputees having problems with socket fit (70). With the first FDA approval for use of OI in the US, significant resources are now being devoted to support key research centres internationally in reducing the risks associated with OI. It is expected that the use of OI will increase over the next decade, offering new possibilities for amputees as the benefits provided by OI are enhanced by more sophisticated, instrumented prosthetic technologies. OI is a classic example of Industry 4.0 embodying the physical, digital and biological in a single entity (31).

### Customer Empowerment

Industry 4.0 is often presented using device-based, hardware and software examples. More difficult to quantify and express are the psychosocial changes occurring in this era, arising from the enthusiastic uptake of the concept of democratization of technology (71). The increase in access to information and communication technologies afforded by Industry 4.0 is supporting shifts in self-image and control, disrupting previous societal organization (72). Debate in society increasingly includes themes of self-empowerment with some persons with disabilities now striving to embrace themselves as they are or to articulate themselves clearly within society, as opposed to hiding their disability (73).

**Figure 3:**
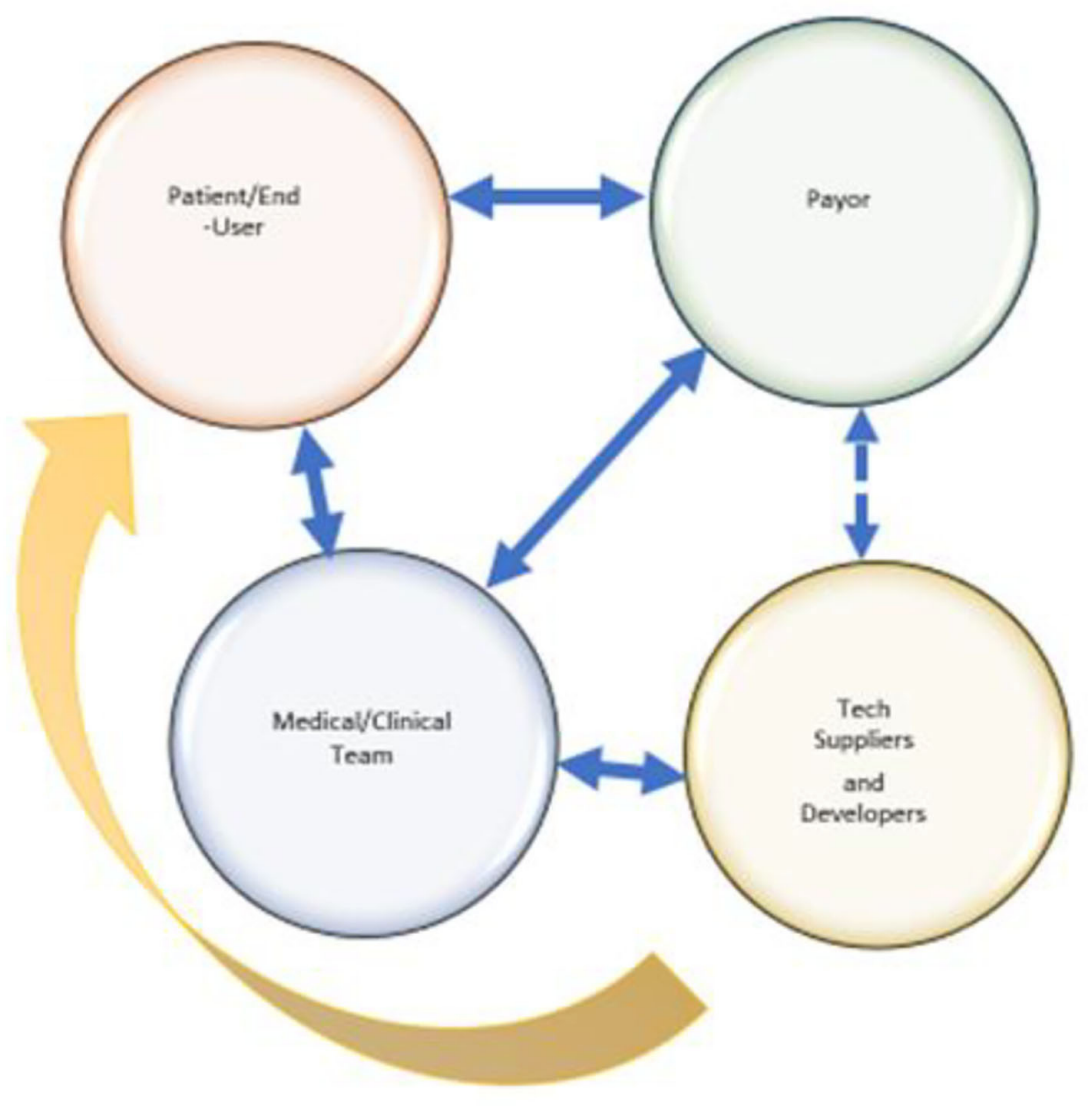
Communication Lines: Established Traditional Communication Lines (Solid Blue), Intermittent Traditional Communication Lines (Dashed Blue), Social Media Enabled (Yellow).

In health care, social media has shifted the power balance between patient and traditional health care provider (74). In the case of durable medical devices, including prostheses, social media has provided the users of prosthetic devices pathways to reach tech developers directly and *vice versa*. Amputees can now communicate their desires and selves directly to engineers and industrial designers, circumventing the traditional “clinical expert” filter who in the past formed a barrier between end-user and engineer (Figure 3).

The result is a range of new approaches to thinking about prosthetic function and design. Fairings are an example of this shift. An aesthetic innovation arising from industrial designers and engineers responding directly to consumer pull, fairings are a non-prescription, add-on product which allows significant personalization and styling of a prosthetic limb. Fairings give amputees the opportunity to express themselves in highly creative and personal ways at relatively low cost and are produced and suppled by new entrants in the prosthetic component sector who use tools such as 3D printing to create their products.

At the high-profile end of this newly created dynamic are social medial influencers such as Aimee Mullins, Amy Purdy and performance artist Viktoria Modesta. These publicly accessible voices speak about concepts such as the prosthesis as a functionally necessary accessory similar to eyeglasses, removing prostheses from the traditional “assistive device” category at the personal and conceptual level, and transforming it into a quasi consumer product. Under this paradigm a prosthesis is no longer is a device that attempts to “make whole,” instead becoming a device which empowers and makes a statement. This approach is currently limited to those with the financial means to purchase multiple or artistically enhanced prostheses and will not necessarily be embraced globally across cultures, or by elderly amputees. It is nonetheless a change in how younger and more active people view both their disability, as well as their prosthesis.

At the other end of this dynamic are amputees who cannot afford any prosthetic device. This group found hope in the potential for additive manufacturing to allow them to gain access to simple, inexpensive prosthetic solutions, creating the first wave of open-source 3D printed hands (upper limb prostheses) that were designed, produced and supplied directly to the amputees, typically by engineering student volunteer groups. The ensuing demand and media attention made it clear that there was an unmet market need that was actively seeking a solution. This first iteration toward that solution did not upend the traditional prosthetic device market, but work in this space continues. A powerful characteristic of additive manufacturing is to allow fast, documentable design iteration and it can be anticipated that efforts by technology developers to create lower cost, customizable designs at the local level will eventually be successful and will have global impact in addressing this unmet market need.

The creation of lower cost, high quality, durable medical devices and health products supports more equitable health care options for all persons at the global level. In low resource settings, developments at the low-cost end of the innovation spectrum have the potential to not only reduce the cost of producing a device for those who cannot afford one, but also by making it possible to move the point of care to the local level. This is of critical importance as the expense of travel to a prosthetic clinic costs more than the device itself, creating an additional barrier. The cost of 3D printing technology continues to decree while the quality of prints increases, in parallel with smart phones become ubiquitous globally and provide access to telemedicine. The intersection of these trends will allow more sophisticated, mobile and affordable care to be delivered close to where it is needed. This vision is consistent with other health care innovations for low resource settings which are now harnessing technology in this way (75).

Under Industry 4.0, low cost or a high level of convenience can no longer be equated with low quality or poor outcome. Disruption has already occurred in other health product categories who have adopted Industry 4.0 enabled approaches with some success. Hearing aids are one such product category, where technology is reconfiguring both the provision process and the business model. At the low-cost end of the market, the FDA has cleared the way for hearing assist devices to be available over the counter for those who cannot afford to, or find it inconvenient to, obtain a traditional hearing aid *via* a hearing aid clinic (76). The palette of options becomes even greater for those with hearing loss, as it is now also possible to do an on-line hearing assessment, to be supplied a hearing aid and to have that hearing aid fitted and tuned *via* web-based provision models. It is no longer necessary to physically go to a clinic for assessment and fitting. Orthodontic bracing systems are a second example. It is now possible to get a series of teeth aligners from storefronts or *via* web-based portals at a lower cost than traditional orthodontia (77). In person assessment followed by regular orthodontist appointments is no longer a necessity. This is not to say that traditional in-person clinical options have been replaced by online models. Many people will continue to prefer in-person models. However, these approaches and business models do provide new options of convenience for some and more equitable access for others. A decade ago both the technological and business approaches embodied in these two examples would have been unthinkable. Similar trends are likely to be seen in prosthetics as well. Indeed, the question must be asked: Why would the purchase of a prosthetic device be any less affordable, accessible, seamless or personalizable?

## Conclusion

**Figure 4:**
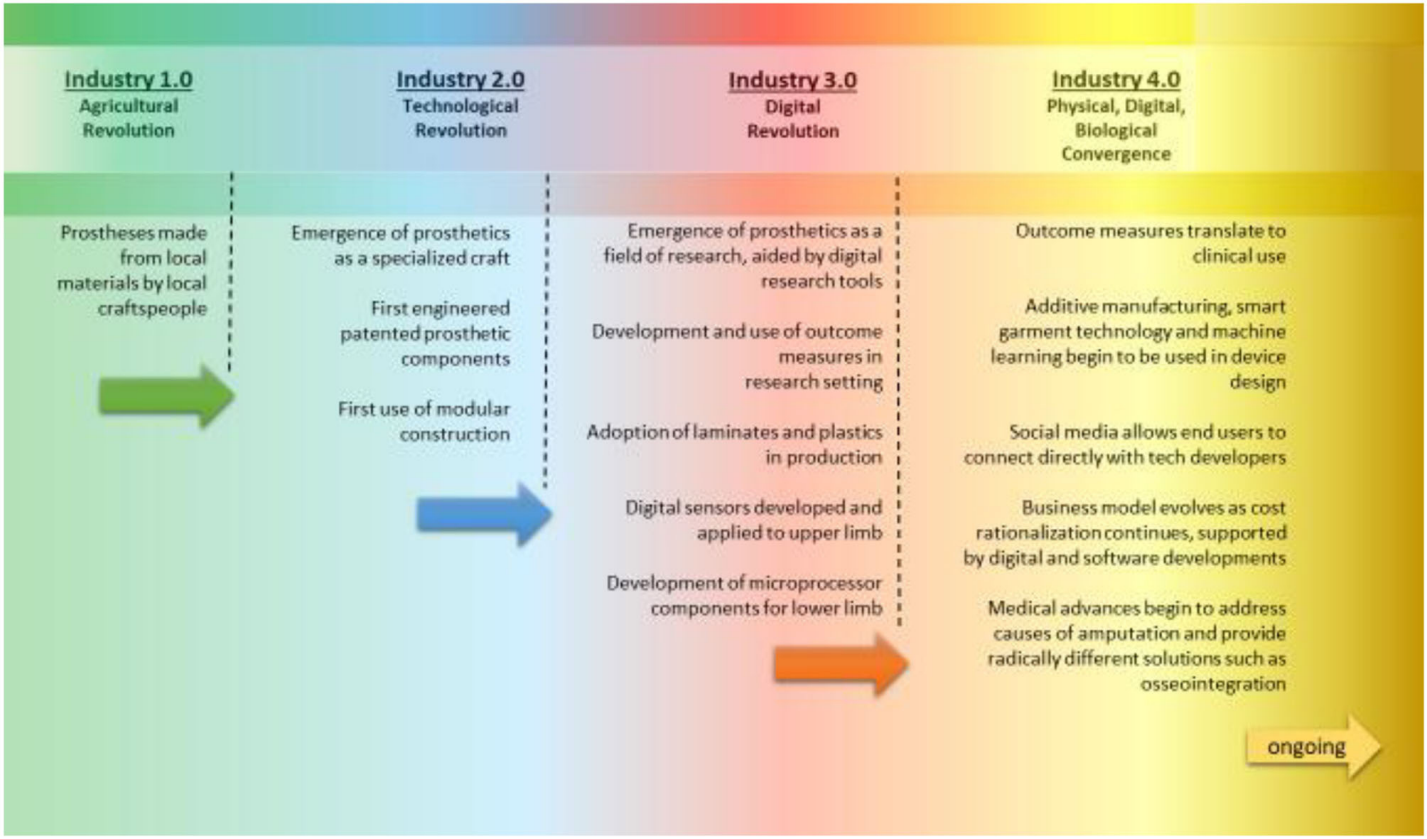
Summary of prosthetic evolution highlights through the eras.

Dr. Stephen Seiler wrote: “History lectures are dangerous: one is forced to compromise completeness for the sake of flow and focus” when presenting a short history of endurance testing in athletes in 2011 (78). This paper does not presume to provide a complete history of technology development in prosthetics. Instead, it intends to identify congruences between the development of technology in society as a whole and advances in technology and practices in prosthetics. A short summary of progress in the prosthetics sector at the transition from one era to another is shown in Figure 4.

It has become commonplace to present technological advances within the framework of a series of Industrial Revolutions beginning in the mid 1700's as societies began to shift from being agriculturally based to industrially based. Over time, further Industrial Revolutions have been identified. Each is defined by a common theme and is discussed as a distinct era but has overlapping start and end points making it more accurate to think of this historical progression as a spectrum where the edges of each period blur into the next. In considering prosthetics using this framework we see that, like most other health sciences, it is a late adoptor of new technology and processes. Prosthetics is a sector that follows, not one that leads, which could explain some of the frustrations and business challenges faced by this relatively small field, when compared to other larger and nimbler sectors. It lacks the critical mass and resources required to take the risks associated with being a leader. At the same time this gives this sector a stability that is lacking in the volatile tech development world, which is of benefit to amputees for whom prosthetic devices are not the “latest gadget” but are critical to their ability to participate in life fully and productively.

The future cannot be predicted, but signposts indicate that prosthetics technology will continue to become more sophisticated, potentially crossing over with robotic or exoskeleton technology. Design and production processes will likely become more automated and will incorporate machine learning and artificial intelligence. With strategic shifts in thinking, Industry 4.0 could allow prosthetic providers to gain sufficient efficiencies within their fee-for-device business model to allow them to focus on providing their clinical services as technology managers, guiding and advising component choice, doing final fittings and ensuring that appropriate function is being provided. Two clear unknowns exist: One is the question of how the business of prosthetics will evolve to become more responsive to increasing consumer expectations while balancing that with payor limitations. The other is how advances in medical treatment options benefitting amputees, but potentially reducing the need for traditional prosthetic solutions, will change prosthetic services and role of the prosthetic provider. Relevance and viability in prosthetics, like all other health sectors, will require an openness to change and flexibility in approach in order for stakeholders to navigate this change in a sustainable way. If done smartly, it will benefits amputees globally. It will also allow prosthetic providers to re-imagine themselves and their role, ideally in a fulfilling way. So where does this leave the sector? In transition, as always.

## Author's Note

SR is an applied researcher with the British Columbia Institute of Technology MAKE+ group. She specializes in evaluation and product development projects in rehabilitation engineering, with a focus on prosthetics and orthotics. In 2013 she and collaborator, Dr. Michael Orendurff, Ph.D. won the Thranhardt Prize for their paper: “Can You Tell Which Foot is Which?.” the first double-blind prosthetic foot evaluation that included community ambulation. She is currently involved in a diverse range of applied and academic research projects, including orthotic aspects of exoskeleton design, 3D printing innovation in foot orthotics, canine assistive devices and is a co-investigator on a study examining Glass Ceilings in Prosthetics and Orthotics. Her interests include how interdisciplinary innovation across a range of sectors is shaping prosthetic design and production practices and business models. She is Co-Editor-in-Chief of the Canadian Prosthetics and Orthotics Journal and Chair of the US Veterans Affairs Rehabilitation Research and Development (RR&D) Subcommittee on Rehabilitation Engineering and Prosthetics/Orthotics.

## Author Contributions

The author confirms being the sole contributor of this work and has approved it for publication.

## Conflict of Interest

The author declares that the research was conducted in the absence of any commercial or financial relationships that could be construed as a potential conflict of interest.

## Publisher's Note

All claims expressed in this article are solely those of the authors and do not necessarily represent those of their affiliated organizations, or those of the publisher, the editors and the reviewers. Any product that may be evaluated in this article, or claim that may be made by its manufacturer, is not guaranteed or endorsed by the publisher.

## Footnotes

^1^ https://stokodesign.com/pages/how-it-works

^2^ https://www.england.nhs.uk/commissioning/spec-services/npc-crg/group-d/d01/prosthetics-review/

^3^ https://www.fda.gov/news-events/press-announcements/fda-approves-prosthetic-implant-above-knee-amputations

^4^ https://www.3dsystems.com/learning-center/case-studies/bespoke-prosthetic-fairings-art-personalized-medicine
